# Prenatal maternal plasma DNA screening for cystic fibrosis: A computer modelling study of screening performance

**DOI:** 10.12688/f1000research.12849.1

**Published:** 2017-10-27

**Authors:** Robert W. Old, Jonathan P. Bestwick, Nicholas J. Wald

**Affiliations:** 1 Wolfson Institute of Preventive Medicine, Barts and the London School of Medicine and Dentistry, Queen Mary University of London, London, EC1M 6BQ, UK

**Keywords:** cystic fibrosis, prenatal screening, DNA sequencing

## Abstract

**Background:** Prenatal cystic fibrosis (CF) screening is currently based on determining the carrier status of both parents. We propose a new method based only on the analysis of DNA in maternal plasma.

**Methods:** The method relies on the quantitative amplification of the CF gene to determine the percentage of DNA fragments in maternal plasma at targeted CF mutation sites that carry a CF mutation. Computer modelling was carried out to estimate the distributions of these percentages in pregnancies with and without a fetus affected with CF. This was done according to the number of DNA fragments counted and fetal fraction, using the 23 CF mutations recommended by the American College of Medical Genetics for parental carrier testing.

**Results: **The estimated detection rate (sensitivity) is 70% (100% of those detected using the 23 mutations), the false-positive rate 0.002%, and the odds of being affected given a positive screening result 14:1, compared with 70%, 0.12%, and 1:3, respectively, with current prenatal screening based on parental carrier testing.

**Conclusions: **Compared with current screening practice based on parental carrier testing, the proposed method would substantially reduce the number of invasive diagnostic procedures (amniocentesis or chorionic villus sampling) without reducing the CF detection rate. The expected advantages of the proposed method justify carrying out the necessary test development for use in a clinical validation study.

## Introduction

Cystic fibrosis (CF) is a severe monogenic autosomal recessive inherited disorder. Over 1,000 mutations have been documented ^1^. In Europe, CF prevalence is about 1 in 2500 live births ^2, 3^, with about 1 in 25 people being a carrier ^4^. In current prenatal screening, parental CF carrier testing identifies couples who are both carriers and offers an invasive diagnostic procedure (amniocentesis or chorionic villus sampling [CVS]) to expectant mothers, one in four of whom will have an affected pregnancy ^5– 8^.

Detecting paternal CF mutations in DNA from maternal plasma as a possible alternative screening method fails because in about 50% of cases the paternal and maternal CF mutations are the same ^9^. The screening method described here overcomes this and does not require parental CF carrier testing. It relies on sequencing and counting DNA fragments, as currently carried out in prenatal DNA screening for Down syndrome ^10– 12^. Unlike carrier testing, which aims simply to detect the presence or absence of mutations in each parent, our method depends on determining the proportion of mutant and non-mutant DNA fragments in maternal plasma. We describe how this proportion can be determined with sufficient statistical precision to distinguish affected from unaffected pregnancies.

The DNA analysis pathway is summarized in Box 1. Plasma DNA comprises short fragments that are typically 100–200bp. Primer pairs are used to hybridise with target sites within about 150bp in the *CFTR* gene for polymerase chain reaction (PCR) amplification of DNA regions that include specified CF mutations. Plasma DNA fragments are tagged with barcodes that provide unique molecular identifiers of each DNA fragment. Such tagging adjusts for and minimises variation in the ratio of mutant to non-mutant DNA sequences that arises from the PCR ^13– 15^. The number of DNA fragments with and without a CF mutation are counted after massively parallel DNA sequencing of the amplified products.

**Box 1.** DNA analysis pathway

Maternal blood 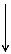 Plasma 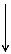 DNA extraction 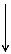 Addition of unique molecular identifiers 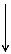 Targeted amplification of DNA regions containing CF mutations 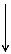 Massively parallel DNA sequencing of targeted regions 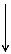 Identify DNA fragments with CF mutations and DNA fragments without CF mutations 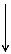 Using the unique molecular identifiers discard excess replicates of individual DNA fragments generated in the amplification step 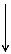 Estimate percentage of fragments with a CF mutation

This concept paper uses results from computer modelling to estimate the number of DNA fragments to be counted at each CF mutation site, and the number of CF mutation sites to be analysed to achieve good expected discrimination between affected and unaffected pregnancies. We then estimate the expected population screening performance of the method.

## Methods

### Estimating maternal plasma distributions of the percentage of targeted CF DNA fragments with a CF mutation

**Figure 1.**
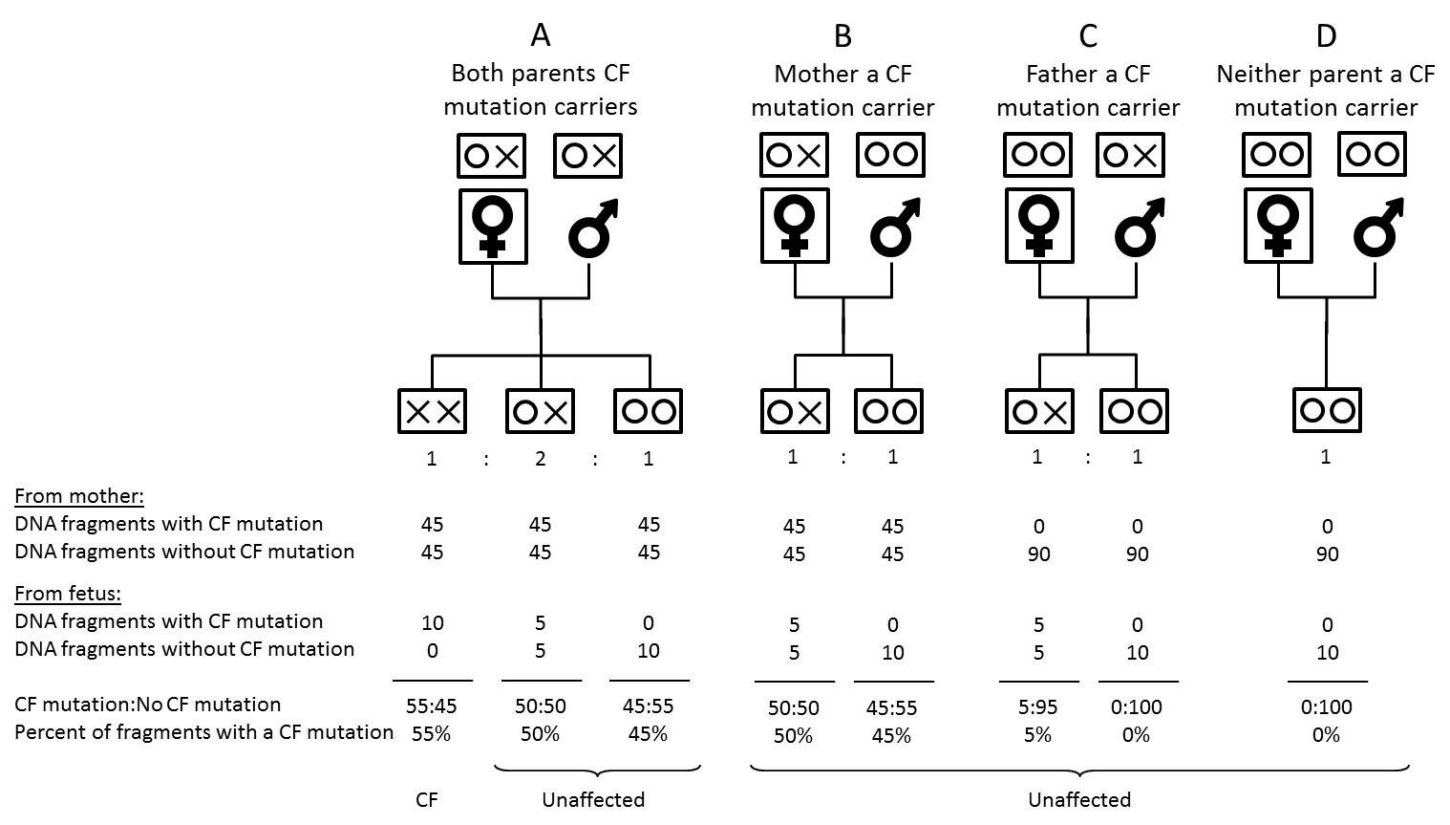
Expected (mean) percentage DNA fragments with a cystic fibrosis (CF) mutation in CF affected and unaffected pregnancies if the fetal fraction is 10%. One or no CF mutation found.

An affected pregnancy is one in which a fetus has a CF mutation on each of the pair of chromosomes 7; other pregnancies, including those with fetuses that are CF carriers, are designated unaffected. For any CF mutation site, the expected (mean) percentage of DNA fragments with a CF mutation in affected and unaffected pregnancies was determined for a given fetal fraction (the proportion of plasma DNA from the placenta). These expected percentages depend on whether the DNA analysis detects two different CF mutations. If not, there are four possible situations, illustrated in Figure 1. In case A in the figure (an affected pregnancy with a 10% fetal fraction), the mother’s plasma will, in expectation, contribute 45% of DNA fragments with a CF mutation and the fetus 10%, i.e. a total of 55%. We estimated the distribution of the percentages of DNA fragments with a CF mutation in affected and unaffected pregnancies for specific fetal fractions using Gaussian distributions with a mean m and a standard deviation $\sqrt{m\times (100-m)/n},$ where n is the number of sequenced DNA fragments in the mutation site. There is one distribution in affected pregnancies with an expected mean of ( *100-ff) / 2* (maternal contribution to all CF fragments) + *ff* (the fetal contribution), where *ff* is the fetal fraction. There are four distributions in unaffected pregnancies; (i) if neither parent is a carrier the mean and the standard deviation are zero, (ii) if the mother but not the fetus is a carrier the expected mean is (100- *ff*)/ 2, (iii) if the fetus is a carrier, with the mutation inherited from the mother the expected mean is (100-ff)/ 2 +ff/2, (iv) if the fetus is a carrier with the mutation inherited from the father the mean is *ff*/2.

Where two different CF mutations are found in the maternal plasma, only the predominant CF mutation (i.e. the more abundant mutation, which is always the one inherited from the mother) is informative. For example, in an affected pregnancy with a 10% fetal fraction, the mother’s plasma will, in expectation, contribute 45% of DNA fragments with a CF mutation and the fetus 10%, half of which is from the father and can be disregarded, i.e. 50% of DNA fragments at the relevant site have a CF mutation. As above, we estimated the distribution of percentages in affected and unaffected pregnancies for specified fetal fractions. The expected mean of the distribution in affected pregnancies is (100-ff)/2 + ff/2 and is (100-ff)/2 if the fetus is a carrier.

The distributions were derived for increasing numbers of DNA fragments counted (the more counted, the larger n, and the smaller the standard error) and for different fetal fractions (the larger the fetal fraction, the more separated the distributions) to determine the minimum counts needed to obtain complete or near complete separation of the distribution in affected and unaffected pregnancies. A positive result was defined as one in which the percentage of DNA fragments with a CF mutation was equal to or greater than a specified cut-off. A screen negative result was one with values below the cut-off.

The above analyses were applied to the 23 CF mutations selected by the American College of Medical Genetics and the American College of Obstetrics and Gynecology ^16^ for parental carrier testing, taking account of their separate prevalence in the pan-ethnic standard population, accounting for an estimated 83.4% of CF carriers.

### Estimating population screening performance

The detection rate (DR, sensitivity: proportion of affected pregnancies with a positive result) was estimated from the proportion of the total area under the distribution of percentage of DNA fragments with a given CF mutation in affected pregnancies equal to or greater than specified cut-off levels, multiplied by the proportion of all CF mutations in the population attributable to the CF mutations in the panel.

The false-positive rate (FPR: proportion of unaffected pregnancies with a positive result), was estimated from the proportion of the total area under the distributions of the percentage of DNA fragments with a given CF mutation in unaffected pregnancies equal to or greater than the specified cut-off levels, multiplied by the proportion of all CF mutations in the population attributable to the CF mutations in the panel. We adjusted for confined placental mosaicism involving trisomy 7 by taking account of its prevalence and the 50% chance that the extra chromosome has the CF mutation ^17– 19^.

The population odds of being affected given a positive result (OAPR) was estimated from the DR divided by the FPR times the prevalence of CF expressed as an odds. The pregnancy prevalence of CF was taken to be 1 in 2500, or 1:2499 as an odds.

## Results

### Distribution of percentage of DNA fragments with a CF mutation

Figure 1 shows the percentage of DNA fragments with a CF mutation in maternal plasma from an affected pregnancy when the two CF mutations are the same (XX) (i.e. only one CF mutation found) according to parental CF carrier status (OX = carrier, OO = unaffected non-carrier). The figure is based on a 10% fetal fraction. If the fetus is affected, the percentage of DNA fragments with the CF mutation in the maternal plasma is, in expectation, 55%. If the fetus is unaffected and is not a carrier, it is 45% or 0% (depending on the parental carrier status). If the fetus is a CF carrier, it is 50% or 5% (again depending on the parental carrier status). In this way, affected pregnancies are distinguished and a result of 55%, which can be statistically separated from the expected 50% or less, defines a positive screening result.

**Figure 2.**
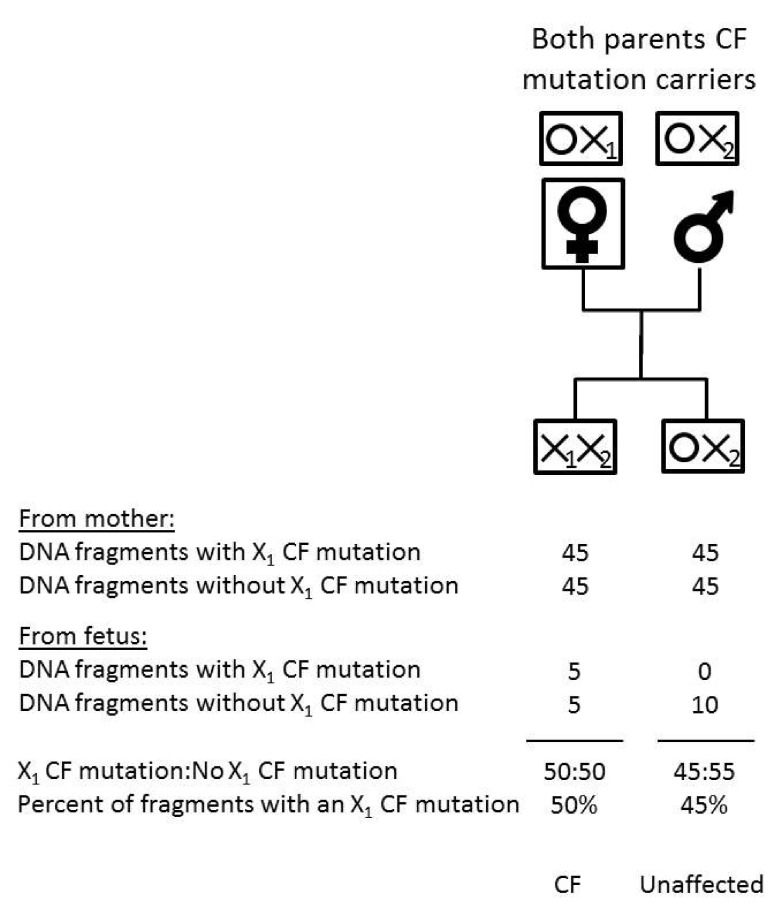
Expected (mean) percentage DNA fragments with the predominant cystic fibrosis (CF) mutation in CF affected and unaffected pregnancies if the fetal fraction is 10% and two mutations are found. Two different CF mutations found.

Figure 2 shows the percentage of DNA fragments with a CF mutation in maternal plasma in an affected pregnancy when the two CF mutations are different (X1 and X2) (i.e. two CF mutations found) according to parental CF carrier status. The fetal fraction is taken as 10%. In maternal plasma the predominant mutation is necessarily from the mother. The expected percentage of CF mutations at the predominant CF mutation site in an affected pregnancy is 50% and 45% in an unaffected pregnancy.

**Figure 3.**
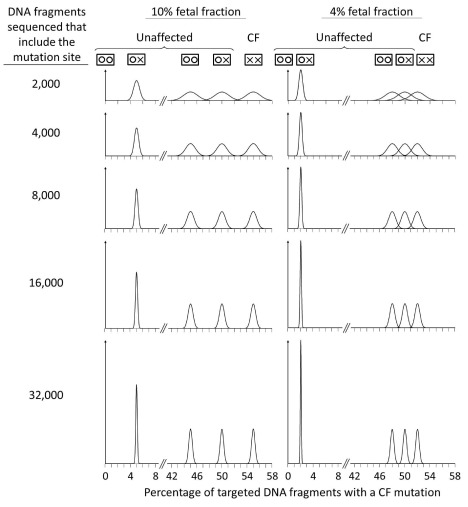
Relative distributions of percent DNA fragments with a cystic fibrosis (CF) mutation in affected and unaffected pregnancies according to fetal fraction and number of targeted DNA fragments sequenced. One or no CF mutation found.

Figure 3 shows the estimated relative distributions of DNA fragments with one or no CF mutation found according to fetal fractions (10%, a typical value, and 4%, a lower limit typically used in prenatal DNA Down syndrome screening) ^20– 22^, the number of DNA fragments sequenced that include the mutation site, and whether the pregnancy is affected or unaffected. With a 10% fetal fraction, counting 8,000 sequenced DNA fragments gives almost complete separation of the relative distributions for the three possible fetal genotypes, with complete (or near complete) discrimination, and consequently a very low FPR. With a 4% fetal fraction, counting 32,000 targeted fragments still gives good discrimination between affected and unaffected pregnancies.

**Figure 4.**
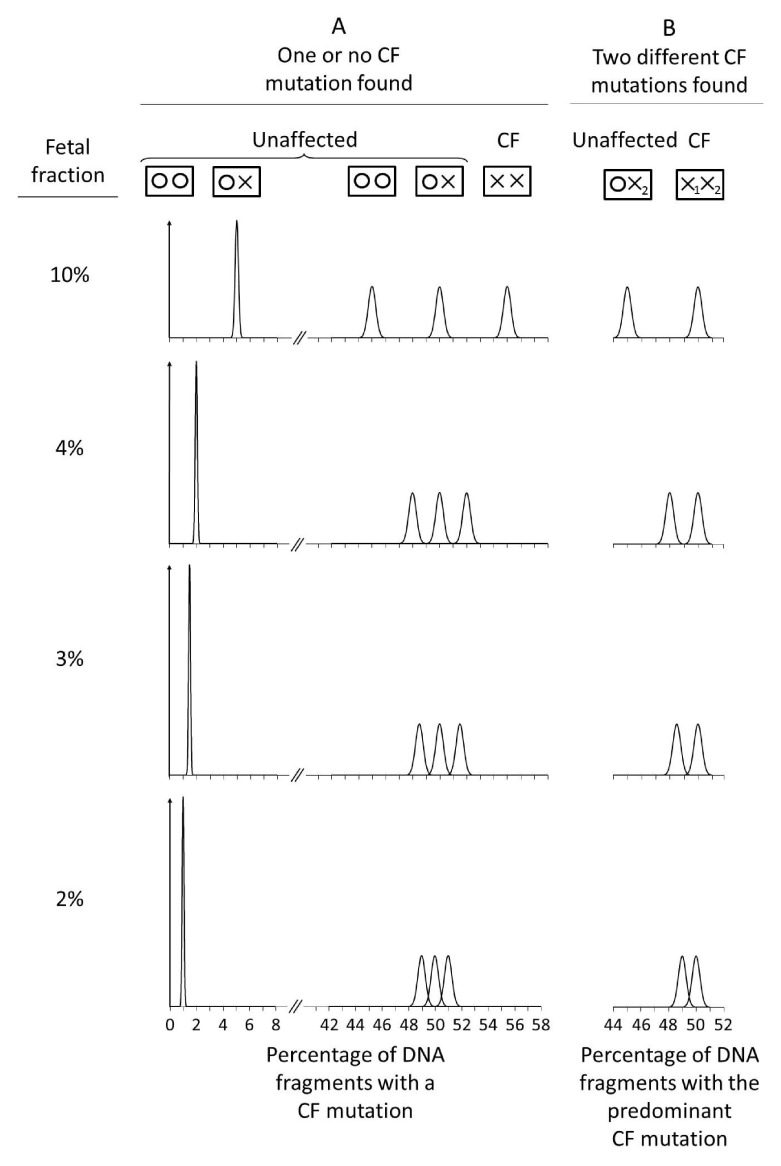
Relative distributions of percent DNA fragments with a CF mutation in affected and unaffected pregnancies according to fetal fraction (32,000 targeted DNA fragments sequenced). ( **A**) One or no CF mutation found; ( **B**) Two different cystic fibrosis (CF) mutations found.

Figure 4 shows the relative distributions of the percentage of DNA fragments with a CF mutation according to fetal fraction if 32,000 DNA fragments are counted. Figure 4A applies if one or no CF mutations are found, and Figure 4B if two different CF mutations are found. Figure 4A shows that with a 3% or greater fetal fraction, there is good discrimination between affected and unaffected pregnancies, but less so with a 2% fetal fraction.

Figure 4B shows that when two different mutations are found the mean for the predominant CF mutation in affected pregnancies is always 50% regardless of the fetal fraction. This is also shown in Figure 2 with a 10% fetal fraction. If the fetal fraction were 4%, the contributions from the mother and the fetus would still sum to 50% i.e. 2% + 48% instead of 5% + 45% as in Figure 2. In an unaffected pregnancy in which the fetus is a carrier, the mean increases towards 50% with decreasing fetal fraction and consequently the cut-off to determine a positive test result is dependent on the fetal fraction. As in Figure 4A, with a 3% or greater fetal fraction, there is good discrimination, but not with a 2% fetal fraction.

### The effect of confined placental mosaicism

Confined placental mosaicism involving trisomy 7, with an estimated prevalence of 0.2% ^17, 18^, has a small influence on the FPR. This effect arises from pregnancies in which the fetus is a CF carrier and has inherited the CF mutation from the mother, and the placental mosaicism is 0XX. This results in more than the expected 50% of DNA fragments with the CF mutation in the maternal plasma. The increase depends on the fetal fraction. Instead of the (unaffected) carrier fetus contributing half fetal fraction to the proportion of mutant fragments in the maternal plasma, it will be two-thirds fetal fraction, i.e. an increase of one-sixth fetal fraction. Given that the mother and fetus are both carriers, and in the estimated 0.1% of pregnancies with confined placental mosaicism of the OXX type, this shift in the distribution of mutant DNA fragments increases the FPR. For example, with a cut-off of 51%, a fetal fraction of 10% and 32,000 DNA fragments counted, the effect of confined placental mosaicism contributes about 0. 002% to the FPR (prevalence of OXX type of confined placental mosaicism (0.1%), times the prevalence of mother and fetus carrier status (2%), times the proportion of CF mutations in the population included in the ACMG panel (0.834) , and making the conservative assumption that nearly all of these false positives would be shifted across the 51% cut-off). Our estimates of screening performance take account of this correction to the FPR. The prevalence of confined placental mosaicism of either the OXX or OOX type (0.1%) is sufficiently low to have a negligible effect on the DR.

### Population screening performance

The 23 CF mutations in the selected panel account for an estimated 83.4% of people with a CF mutation, so the maximum CF DR (proportion of CF pregnancies detected) is 70% (83.4% × 83.4%), because for a fetus to be affected it must have two CF mutations, one from each parent, assuming random mating.

Table 1 shows the estimated screening performance according to the screening cut-off (expressed as the percentage of targeted DNA fragments with a CF mutation), and fetal fraction using the 23 CF mutation panel. The cut-off of choice is 51% when one or no CF mutation is found in the maternal plasma sample. When two CF mutations are found in the maternal plasma, the cut-off will vary according to fetal fraction (eg. 46% with a 10% fetal fraction or 49% with a 4% fetal fraction. Provided the fetal fraction is 3% or more, a DR of 67–70% (limited mainly because of the number of mutations used in the test, not by the DNA analysis) can be achieved with a very low FPR (≤ 0.002%). Counter-intuitively, the OAPR increases with decreasing fetal fraction. This arises as a result of two competing effects; an underlying decrease in the OAPR with decreasing fetal fraction and a diminishing false-positive rate due to the placental mosaicism, the latter dominating. Even in the presence of placental mosaicism a low fetal fraction is a disadvantage because of the reduced detection rate. Table 1 also shows that the DR is reduced to 35% with a 2% fetal fraction, setting a practical lower limit of 3%; <1% of pregnancies have a fetal fraction <3% ^20^. The 70% DR shown in Table 1 requires the use of 23 CF mutations in the test and an estimated 736,000 targeted DNA fragments need to be counted (23 mutations × 32,000 fragments per mutation).

**Table 1.** Screening performance according to cut-off of percentage of targeted DNA fragments with a CF mutation and fetal fraction (32,000 DNA fragments sequenced per mutation in the 23 CF mutation panel) using the 23 common mutation panel recommended by the American College of Medical Genetics ^16^.

	Cut-off (% of DNA fragments with a CF mutation)				
Fetal fraction (%)					
	1 CF mutation found	2 CF mutations found *	DR (%)	FPR (%)	OAPR
20	50	40	70	0.8	1:29
	51	41	70	0.002	14:1
	52	42	70	0.002	14:1
10	50	45	70	0.8	1:29
	51	46	70	0.002	14:1
	52	47	70	<0.001	130:1
4	50	48	70	0.8	1:29
	51	49	70	<0.001	140:1
	52	50	35	<0.001	>1000:1
3	50	48.5	70	0.8	1:29
	51	49.5	67	<0.001	420:1
	52	50.5	3	<0.001	>1000:1
2	50	49	70	0.8	1:29
	51	50	35	<0.001	860:1
	52	51	<0.1	<0.001	>1000:1

*Cut-off to yield same screening performance as when only 1 mutation is found. DR=detection rate; FPR=false-positive rate; OAPR=odds of being affected given a positive result If FPR < 0.001% OAPR is precise unless over 1000:1.

## Discussion

Prenatal DNA screening for CF has a higher predicted screening performance than conventional screening based on parental carrier testing. The improved screening performance is based on maintaining the DR achieved using a given parental carrier testing CF mutation panel but with a 60 times lower FPR, 0.002% compared with 0.12% (prevalence of carrier couples is 4% × 4% = 0.16%, assuming random mating and, among these, 75% are false-positive (0.16% × 0.75 = 0.12%)).

**Figure 5.**
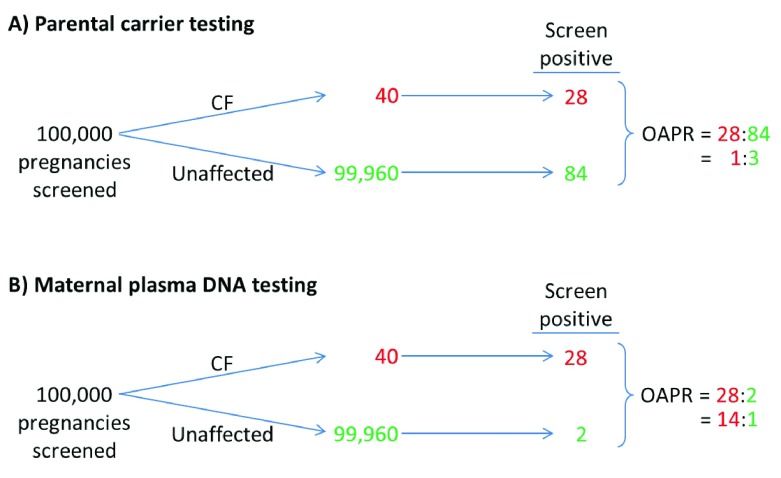
Illustration of prenatal screening for cystic fibrosis (CF) based on **A** parental CF carrier testing using the 23 common mutation panel recommended by the American College of Medical Genetics ^16^ and **B** screening using the maternal plasma DNA with the same 23 CF mutations (fetal fraction 10%, 51% cut-off if one CF mutation found, 46% if 2 found [see Table 1]).

Figure 5 compares prenatal CF screening based on parental carrier testing with plasma DNA screening. The figure shows its clinical advantage in achieving a much lower FPR and hence a much higher OAPR, 42 times higher (14:1/ 1:3) than parental carrier testing. An estimated 98% of invasive diagnostic tests in unaffected pregnancies are avoided, without loss of detection (82/84 in Figure 5).

The selection of a screening cut-off of targeted DNA fragments with a CF mutation should balance maximizing the DR, minimizing the FPR, and achieving an acceptably high OAPR. A cut-off of 51% is reasonable ( Table 1). This cut off, with a 10% fetal fraction, achieves an expected 70% DR with a FPR of 0.002%. A higher cut-off of 52% results in a loss of detection with low fetal fractions even though the FPR is decreased. A 50% cut-off retains detection but at the cost of a much increased FPR.

Plasma DNA screening is simpler than parental carrier testing because it only requires a maternal plasma sample. It also avoids a problem with parental carrier based screening that arises when the biological father (but not the assumed father) is a carrier, and the pregnancy is affected. The rate of non-paternity varies among populations; in one study it was 2% ^23^.

Plasma DNA screening treats each pregnancy as a fresh screening opportunity, which is similar to prenatal screening for neural tube defects or Down syndrome. The screening aims to identify an affected pregnancy. There is no intention to identify carriers, which is a benefit from a screening perspective, because almost all carriers will never have an affected pregnancy. Being a carrier is of minor or no medical consequence. The fact that the disorder being screened for is inherited is, from the screening perspective, irrelevant. This approach has the advantage of screening women who have a pregnancy with a different partner without the need to retrieve a report on her previously determined carrier status, and if a carrier, determining the carrier status of her new partner.

Unique molecular identifiers are needed to overcome random error in DNA fragment amplification. With the use of unique molecular identifiers amplification, sequencing, and counting can be corrected for under- and over-amplification ^13– 15^. Plasma DNA sequencing as described here, using unique molecular identifiers, overcomes the limitation associated with digital PCR ^24^ that does not quantify mutant and non-mutant DNA sequences sufficiently accurately to reliably distinguish affected from unaffected pregnancies. Whole-genome sequencing could overcome the digital PCR limitation, but is impractical because of the cost of unnecessary sequencing of most of the genome ^25^. Our paper provides a potentially practical cost-effective solution, the screening algorithms needed, and computer modelled estimates of population screening performance.

**Figure 6.**
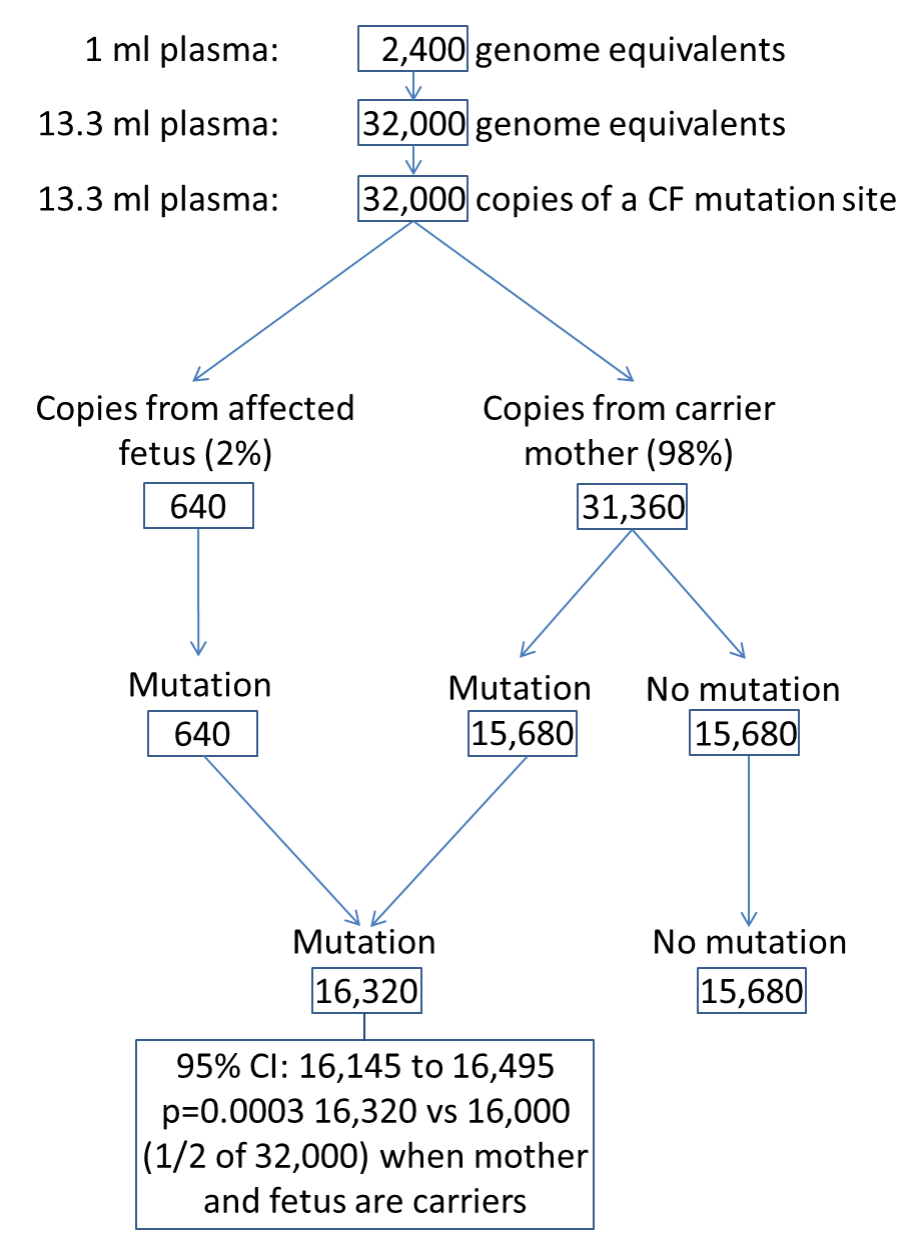
Illustration of number of DNA fragments at a CF mutation site in a pregnancy where the fetus is affected and the fetal fraction is 2%, compared with the number in a pregnancy where both the mother and fetus are carriers (one CF mutation is found).

To obtain the 32,000 DNA fragments containing a CF mutation site needed for the test if the fetal fraction is as low as 2% requires about 13ml of plasma (i.e. about 30ml blood), because 1ml typically contains about 2400 haploid (single duplex DNA strand) whole genomic equivalents, each of which has one copy of each CF mutation site ^26, 27^ (32,000/ 2,400). Figure 6 illustrates how 13.3ml of plasma provides enough target sites to distinguish an affected fetus from a maternally derived fetal carrier fetus (one CF mutation found). There would be enough blood for a DNA screening test for trisomy 21, 18, and 13, as well as CF. The plasma DNA test should cost little more than about half the cost of a Down syndrome DNA screening test because sequencing accounts for about half the test cost, and the sequencing cost of the CF test is reduced to about a tenth or less of that required for Down syndrome screening

As the number of CF mutations used in the test increases, the number of DNA fragments to be counted also increases without, however, requiring a larger plasma sample because many CF mutation sites can be amplified simultaneously ^14^. Using 23 CF mutations, 736,000 (23 × 32,000) DNA fragments need to be counted. Adding prenatal CF screening to DNA-based screening for Down syndrome is feasible and involves only an extra step to amplify the selected CF DNA sites prior to sequencing.

The number of CF mutations tested limits the overall DR. Some current parental carrier testing uses more CF mutations than the 23 used here; one programme uses a 106 mutation panel, accounting for an estimated 91% of people with a CF mutation in the population specified. This yields an 81% DR (91% × 91%). A panel of about 100 mutations should be feasible with maternal plasma DNA screening; however, the incremental increase in detection with increasing number of CF mutations is very small. Any panel used could be modified according to the ethnic distribution of the population screened. Maternal plasma DNA screening using full exon sequencing (which would include all CF mutations) may be a future option, but there may be a limit to the number of DNA regions that can be amplified.

While plasma DNA screening does not rely on parental CF carrier detection, it still has a useful role to play where CF carrier testing has already been established. The relevant CF mutations would be known from parental testing and, provided the parental CF mutations were included in the method, all affected pregnancies would be identified and amniocentesis or chorionic villus sampling would be avoided in nearly all unaffected pregnancies. Therefore, about 3/4 women would avoid an invasive diagnostic procedure.

The proposed method could be used in a two-step screening procedure in which the CF carrier status of the mother is first identified and then, if she is a carrier, the described method adopted. This may have cost savings as, if the mother is not a carrier, there is no need to proceed with further testing. Also, it would lend itself to other autosomal recessive disorders. The method described here need not, therefore, be limited to CF.

Although *de novo* CF mutations are rare, the proposed method detects them, provided they are included in the specified mutation set. Interpretation of test results if the fetus has a *de novo* mutation applies as set out in this paper, with three exceptions: (i) mother is not a carrier but the father is a carrier - in this situation the percentage DNA CF fragments is on average 10% if the mutation is the same as the father’s mutation or 5% if it is different (assuming a 10% fetal fraction); (ii) mother and father are carriers, the father carries a different CF mutation, the *de novo* mutation is the same as the father’s mutation and the fetus inherits the father’s mutation - in this situation the affected pregnancy would be missed; (iii) mother and father are carriers, the father carries with a different mutation, the fetus has a mutation that is different from both and the fetus inherits the father’s mutation - in this situation the affected pregnancy would be detected by the presence of three different CF mutations. While in one of these situations an affected pregnancy would be missed, the method achieves a higher level of detection than screening based on parental carrier testing, which misses all cases with a *de novo* mutation except for those where both parents are carriers.

## Conclusion

Prenatal maternal plasma DNA screening for CF has an estimated screening performance substantially higher than current screening based on parental carrier testing. While amniocentesis is still required for the diagnosis of CF, the proposed method means that nearly all amniocenteses would be performed in affected pregnancies, without reducing the DR. The overall expected advantages are sufficiently large to merit developing the test for use in routine screening practice and evaluation in a clinical validation study.

## Data availability

The data referenced by this article are under copyright with the following copyright statement: Copyright: © 2017 Old RW et al.

All data analysed in this study are cited through the article.
