# Supplementary material for: Prenatal maternal plasma DNA screening for cystic fibrosis: A computer modelling study of screening performance
Source: F1000Res. 2017 Oct 27;6:1896. [Version 1] doi: 10.12688/f1000research.12849.1 (PMC5680537; doi:10.12688/f1000research.12849.1)
Supplement: Peer review history

# Peer review: Referee response for version 1

**DOI:** 10.5256/f1000research.13924.r27536

**Published:** 2017-11-08

**Authors:** Ioannis Prassas

**Affiliations:** 1 Department of Pathology and Laboratory Medicine, Mount Sinai Hospital, Toronto, ON, Canada

A novel study which introduces a novel method for prenatal detection of CF. It is based on the direct quantitative amplification of the CF gene to determine the percentage of targeted DNA fragments (CF mutation sites) in maternal plasma. Avoiding the need for standard parental carrier testing, the proposed procedure is positioned to reduce the number of invasive diagnostic procedures (amniocentesis or chorionic villus sampling) without compromising the total CF detection rate. 

 The study is well-designed and the results are carefully interpreted. The conclusions drawn are adequately supported by the results. The few associated limitations of the proposed technology are properly highlighted by the authors.

I have read this submission. I believe that I have an appropriate level of expertise to confirm that it is of an acceptable scientific standard.

# Peer review: Referee response for version 1

**DOI:** 10.5256/f1000research.13924.r27399

**Published:** 2017-11-02

**Authors:** James E Haddow

**Affiliations:** 1 Department of Pathology and Laboratory Medicine, Brown University, Providence, RI, USA

This concept paper describes a methodology in maternal plasma, aimed at detecting homozygous CF mutations in free fetal DNA. The philosophy underlying this proposal places emphasis on screening directly for fetal CF, thereby avoiding first level screening that focuses on screening to identify CF carriers when both parents are found to be heterozygous for individual mutations. In this latter circumstance, the fetus would have a 1 in 4 risk of having CF. Invasive testing (amniocentesis or chorion villus sampling) would provide the definitive answer. The methodology described here, which detects the fetus directly, also requires invasive testing for confirmation but avoids 3 out of 4 invasive procedures required by parental carrier screening.

 The present concept is a product of expertise involving several disciplines, including molecular biology, population screening (with emphasis in pregnancy), and biostatistics. Description of the methodology is plausible, including limitations resulting from low percentage of free fetal DNA in a given sample, and volume of maternal blood sample necessary for adequate numbers of DNA fragments. This concept has been extensively thought out, including potential applications in various screening scenarios. Although not explicitly stated, the methodology appears ready for testing as a proof of concept. Assuming success, this could then serve as a prelude to determining how most appropriately to introduce implementation.

I have read this submission. I believe that I have an appropriate level of expertise to confirm that it is of an acceptable scientific standard.
